# Experiment Study on Damage Properties and Acoustic Emission Characteristics of Layered Shale under Uniaxial Compression

**DOI:** 10.3390/ma16124317

**Published:** 2023-06-11

**Authors:** Binke Chen, Zhiqiang Zhang, Qingnan Lan, Zheng Liu, Yinjun Tan

**Affiliations:** 1 School of Civil Engineering, Southwest Jiaotong University, Chengdu 610031, China; becker91910315@gmail.com (B.C.); lqn0929@126.com (Q.L.); leonviho@163.com (Z.L.); yinjuntan_1993@my.swjtu.edu.cn (Y.T.); 2 Key Laboratory of Transportation Tunnel Engineering, Ministry of Education, Southwest Jiaotong University, Chengdu 610031, China

**Keywords:** gently tilt-layered shale, AE characteristic parameters, uniaxial compression, time varying changes, failure modes

## Abstract

The gently tilt-layered shale displays anisotropic behavior and includes structural planes that cause the rock to exhibit weakened features. As a result, the load-bearing capacity and failure mechanisms of this type of rock differ significantly from those of other rock types. A series of uniaxial compression tests were performed on shale samples from the Chaoyang Tunnel to investigate damage evolution patterns and typical failure characteristics of gently tilt-layered shale. An acoustic emission testing system was incorporated to analyze the acoustic emission parameters of the shale samples during the loading process. The results indicate that the failure modes of the gently tilt-layered shale are significantly correlated with the structural plane angles and water content. The shale samples gradually transition from tension failure to tension-shear compound failure as the structural plane angles and water content increase, with an increasing level of damage. The maximum values of AE ringing counts and AE energy for shale samples with diverse structural plane angles and water content are reached near the peak stress and serve as precursors to rock failure. The primary factor influencing the failure modes of the rock samples is the structural plane angle. The precise correspondence between the structural plane angle, water content, crack propagation patterns, and failure modes of gently tilted layered shale can be captured by the distribution of the RA-AF values.

## 1. Introduction

A rock mass is a geological body with discontinuity, heterogeneity, and anisotropy composed of various types of rock containing planes of low structure within a certain engineering range. In the natural environment, rock masses typically possess natural defects such as fractures and joints [1]. The presence of these discontinuities significantly affects the mechanical performance of rock masses and alters their mechanical behavior [2,3]. These gently sloping masses of layered rock have significant anisotropy and structural plane weakening characteristics, which causes its mechanical characteristics and mechanism of failure to differ significantly from other rock masses. A number of built and under construction, gently sloping, bedded rock mass tunnels have experienced severe disease events, and there is a pressing need to study the law of damage evolution in gently sloping bedded rock mass as well as the development of rock mass cracks.

At present, the employment of acoustic emission (AE) technology is predominantly observed in the domain of geotechnical engineering for the investigation of acoustic emissions characteristics, as well as the strength and brittle damage of rocks under varying rock types and loading circumstances [4,5]. As a kind of nondestructive testing (NDT) method, the AE technique provides an efficient means of detecting rock damage processes, which facilitates the determination of the damage evolution of internal micro-structures in rocks, further allowing for an accurate forecast of brittle failure in structures [6,7]. Acoustic emission from rocks is the elastic wave released by the propagation of cracks and primary defects in the materials of the rock during stress, as well as the incubation, evolution, and fracture process of new microcracks. This phenomenon contains a great deal of information about the internal failure process of rocks [8,9]. An AE signal constitutes a transient elastic wave and has been utilized for monitoring and predicting rock stability and shatter in mining pillars. The earliest AE techniques have been specifically employed for this purpose. The spectral features of the AE signal can effectively determine the mechanical properties, structure, and stress state of the rock [10]. In fact, the characteristics of the acoustic emission signals are significantly correlated with the material fracture caused by loading. By analyzing their characteristic values, the variation patterns of internal cracks in rocks can be accurately obtained, the crack properties can be judged, and macroscopic damage can be predicted [11,12]. Many researchers use characteristic metrics such as the counting of AE events, the scaling of the AE count rate with the stress–strain relationship, and the source localization of events to study fracture characteristics such as crack propagation, source localization, and quantitative analysis of damage in rocks and other solid materials [13,14,15,16,17,18]. Through the exploration of characteristic features of acoustic emission activity, Li et al. investigated the influence of free water content on the loading capacity and failure mechanisms of concrete under uniaxial compression conditions [19]. Chen et al. measured the AE characteristics of travertine in Jiuzhaigou during triaxial compression tests and established a comprehensive failure model that considers both characteristic modulus and AE features [20]. Li et al. examined the AE signal time series variation law of siltstone during uniaxial compression based on the information entropy weighting theory [21]. Jiang et al. conducted fracturing tests on the natural coal-rock block specimens and investigated the crack propagation behavior of the specimens under different stress conditions and fracturing media [22]. Dong et al. thoroughly examined the accuracy of the RA-AF approach (RA is the rising time-amplitude ratio; AF is the average frequency) in determining the different types of microcracks in rocks considering measurement errors and propagation losses of AE parameters [23].

Furthermore, several scholars have observed notable dissimilarities in the AE properties of rocks that have been exposed to varying loading rates during a sequence of laboratory tests. Subsequently, they carried out extensive experimental investigations aimed at examining the effects of loading rate on the AE characteristics of these rocks [24,25,26,27,28]. Additionally, waveform parameters that are linked to the generation of AE events, including but not limited to the number of rings, energy, peak magnitude, rise time, and event duration, can furnish valuable insights for further exploration into the mechanisms associated with macroscopic deformation and rupture of rocks. A number of scholars have conducted several indoor experiments to study the relationship between macroscopic mechanical features such as stress–strain, crack propagation, and the aforementioned parameters of AE waveforms during the tensile compression and unloading processes of rocks. Zhao et al. [29] conducted tests on granite using both uniaxial and triaxial compression methods. In their study, they investigated the connection between the AE characteristics and the macroscopic failure modes of the rock. Following their work, there has been a renewed interest in examining the relationship between the frequency amplitude of AE signals and the characteristics of rock burst stages [30]. This correlation was explored by Wasantha et al. [31] and Wang et al. [32] through rock compression tests, and the relationship between the rock mechanical behavior and the AE energy characteristics is discussed. The study of Khanzhin et al. [33] further validated the accuracy of using AE to analyze the deformation and fracture of brittle materials. On this basis, Nikulin et al. [34] also proposed necessary conditions for the use of acoustic emission for the characterization of damage. Chen et al. [35] studied the link between the parameters of the AE features and the failure mechanism of the rock mass containing a rock bridge and the spatial crack evolution process based on the direct shear testing of the rock bridge. Based on acoustic emission theory, Wu et al. [36] explored the damage characteristics of salt rock by uniaxial creep under quasi-static loading conditions. The acoustic emission characteristic parameter (RA-AF) was proposed by Tang et al. [10] to predict interface instability via the direct interface shear test of rock media with binary mortar. Yang et al. [37] studied the AE features of confining pressure tests of coal and rock with triaxial discharge. Their findings suggest that the HURST index of the AE time series can serve as an indicator for assessing unloading and fracture when it is found to be equal to 0.8. Li et al. [38] investigated the AE energy, the count, and the peak frequency of four natural rock samples in hydraulic fracture tests. The experimental results provide a good explanation for the laws of rock crack propagation and evolution. Zhang et al. [39] explored the process of tensile deformation and failure of rocks from a microscopic perspective based on quantitative analysis of the acoustic emission waveforms and provided criteria for the selection of design parameters within rock engineering. Wang et al. [40] studied the AE characteristics of rock samples with a variety of crack angles under uniaxial compression conditions, in addition to discussing the deformation and fracture mechanisms of rock damage.

Current research has made significant achievements in the development and application of acoustic emission monitoring technology in rock. Unfortunately, there is a dearth of research on the mechanical properties of acoustic emissions, three-dimensional spatial evolution of microcrack initiation, propagation, and connection processes, as well as the damage fracture mechanism of rock samples with different structural plane angles and water contents. This study employs AE counting to conduct uniaxial compression tests on precast rock samples featuring different structural plane angles (0°, 15°, and 30°) and varying moisture contents (dry, natural, and saturated). The entire rock loading process’ AE signal is captured through the employment of a fully digital acoustic emission monitoring system. Subsequently, the AE parameters’ time-varying characteristics and crack propagation mechanism during the rock failure process are evaluated, and the damage deformation and failure mechanism of layered shale at gentle inclinations under uniaxial compression conditions are investigated. The research findings are expected to enhance our comprehension of rock failure mechanisms and to propose sound pre-cursor criteria for rock failure. Additionally, a benchmark for the development of on-site monitoring and prediction technology for the emission of acoustic rock and the assessment of rock stability is provided.

## 2. Tunnel Overview and Engineering Geology

The Chaoyang Tunnel of the Guinan Passenger Dedicated Line is a tunnel in the section from Dushan South to Libo in Guizhou. It has an inlet of DK159+802 and an outlet of DK172+536, with a total length of 12,734 m and a maximum embedding depth of approximately 432 m. The inlet is adjacent to the Di’e River double-track bridge, and the outlet is connected to the proposed roadbed. The longitudinal slope of the line is a single downhill, with a 25‰ downhill of 12,598 m at the inlet end and a 22.7‰ downhill of 136 m at the outlet end.

**Figure 1:**
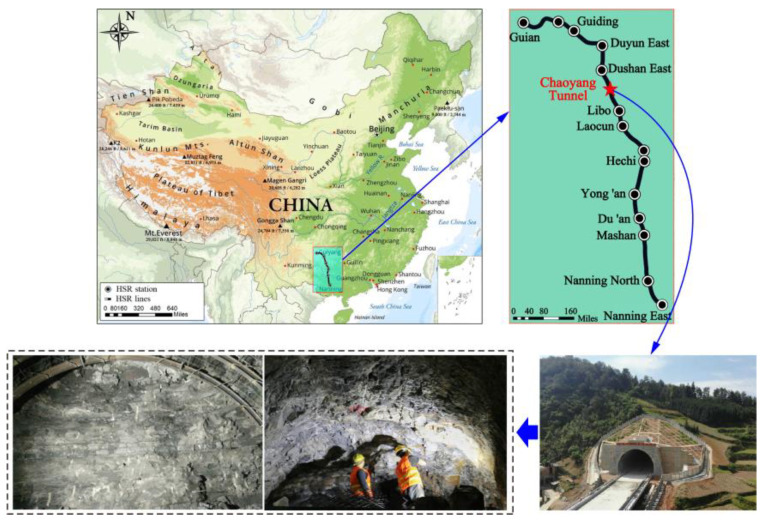
Sketch map of the topography and landforms of the study area.

The research area belongs to a landform of medium- to low-mountain and karstic ridge groups, with significant fluctuations in the natural terrain and slopes typically varying between 20~55°, with locally formed steep cliffs. Based on the regional data and the on-site survey, there are a total of three developed faults within the survey area, namely Di’e 1 # normal fault, Di’e 1 # reverse fault, and the Chaoyang reverse fault. Groundwater is available in a variety of types, with the corrosivity of sulphuric acid to concrete, and the level of erosion is H1. The surrounding gently sloping section of bedded rock in the tunnel has an overall length of 4.27 km, with a burial depth of approximately 200~370 m. According to the geological profile of the “Regional Geological Survey Report”, this section of the tunnel passes for the most part through the old section of the Lower Carboniferous Datang Stage (C1d1). The lithology of the strata comprises interbedded argillaceous limestone, carbonaceous shale, and sandstone formations, exhibiting predominantly gray, light yellow, and dark gray hues. The layers are moderately thick with occasional thin interlayers, characterized by clayey cementation. The sandstone and argillaceous limestone formations exhibit a moderate thickness, while the shale formations are primarily thin and laminated. The carbonaceous shale is characterized by a foliated and scaly structure. The focus of this research article pertains to the shale formations within the Chaoyang Tunnel. The terrain and topography of Chaoyang Tunnel are shown in Figure 1.

## 3. Uniaxial Compression with AE Measuring

### 3.1. Specimen Preparation

**Figure 2:**
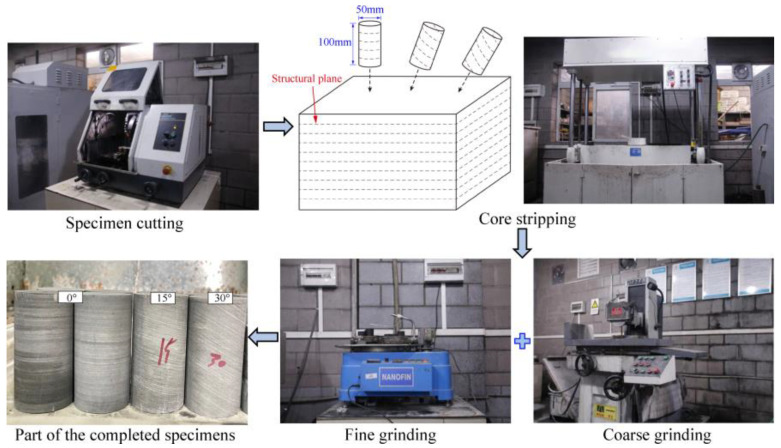
Specimen preparation process.

The gently sloping layered shale rock masses were obtained from the Chaoyang Tunnel under construction, with a burial depth of 240 m at the sampling point. The gently sloping layered shale samples with varying water contents were prepared in accordance with the Regulation for testing the physical and mechanical properties of rock (DZ/T 0276.10-2015) [41]. The preparation process for the samples is as follows:

(1) Coarsely cut. Cut the large, gently sloping blocks of layered shale from the face of the tunnel into square blocks.
(2) Core. Place the corer and drill rock core positions with a diameter of 50 mm and structural plane angles of 0°, 15°, and 30° on the square blocks of shale.
(3) Finely cut and polish. The production of a typical cylindrical sample for experimental testing purposes involves the acquisition of a rock core, which is subsequently cut and polished to achieve a standard specimen with a height of 100 mm and a diameter of 50 mm. This process is illustrated in Figure 2. To control for standard sample size error within ±0.5 mm, the end-face parallelism error should be controlled to within ±0.02 mm.
(4) Natural test preparation. Directly entrap the standard sample in cling film as a natural sample.
(5) Dry sample preparation. In order to prepare a dry sample, the standard sample is dried at 105 °C in a dryer for 24 h.
(6) Preparation of saturated samples. To make a saturated sample, the dry sample is placed in a forced saturator under a vacuum for 24 h.

After measurement, the moisture content of the dry sample, natural sample, and saturated sample is 0%, 2.4%, and 5.3%, respectively.

### 3.2. Experimental Equipment and Method

The method for determining the location of acoustic emission involves analyzing the time difference between P(S) waves detected by probes placed at varying locations. The Geiger algorithm is utilized to derive the source location of acoustic emission within the stone, thus enabling the identification of internal damage and microcrack propagation within the rock. This study aims to identify the time-dependent characteristics of acoustic emission parameters and the propagation of microcracks during the stress, strain, and failure process of rock samples with differing structural plane angles and water contents, as determined through uniaxial compression rock-breaking tests.

**Figure 3:**
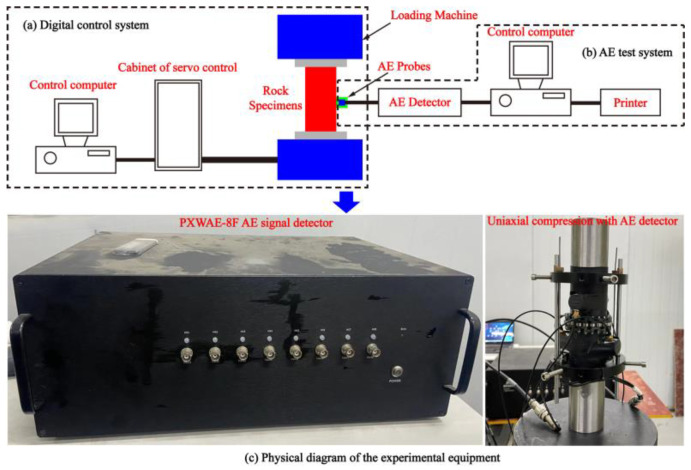
Acoustic emission system of uniaxial compression.

Figure 3a,b show that the test equipment consists of a charge control system and an AE monitoring system. It is a fully digital computerized automatic control system capable of tracking, recording, and drawing load displacements and stress–strain curves in a synchronized manner. The MTS851 (Metus Industrial Systems Co., Ltd., Shanghai, China) hydraulic servo rocker mechanical testing machine utilizes axial displacement control for loading, with a loading rate of 1 × 10^−3^ mm/s. It offers an axial load range of 0 to 4600 kN, a confining pressure range of 0 to 140 MPa, a pore water pressure range of 0 to 70 MPa, a hydraulic permeability pressure difference range of 0 to 2 MPa, and a hydraulic source flow rate of 31.8 L/min.

To eliminate any potential interference signals that may arise during the initial loading phase, a process of pressure polishing and the application of petroleum jelly was conducted at both ends of the specimen before test loading. During the loading phase, the selection of the acoustic emission instrument PXWAE-8F (Changsha Pengxiang electronic Technology Co., Ltd., Changsha, China) allowed for synchronous monitoring of acoustic emission signals from the gently tilt layered shale, as depicted in Figure 3c. The PXWAE-8F AE collector is a high-speed acoustic emission acquisition instrument that utilizes 18-bit sampling precision and real-time acquisition at a sampling rate of 30 million samples per second. It offers a transmission bandwidth of 1 kHz to 5 MHz. The PXWAE-8F employs multi-channel synchronized acquisition, ensuring high data integrity and accuracy. The sampling interval of the AE monitoring system is set to 50 μs, and the range of frequencies acquired by the PXWAE-8F AE collector during the uniaxial compression test is 10^2^~10^4^ Hz. To ensure that the test exclusively measured major acoustic emission events resulting from the uniaxial compressive failure of the rocks, a signal trigger level of 3.6 mV was selected to minimize any potential interference from noise.

## 4. Experimental Results and Analysis

### 4.1. Uniaxial Compression Mechanical Characteristics of Layered Shale

**Figure 4:**
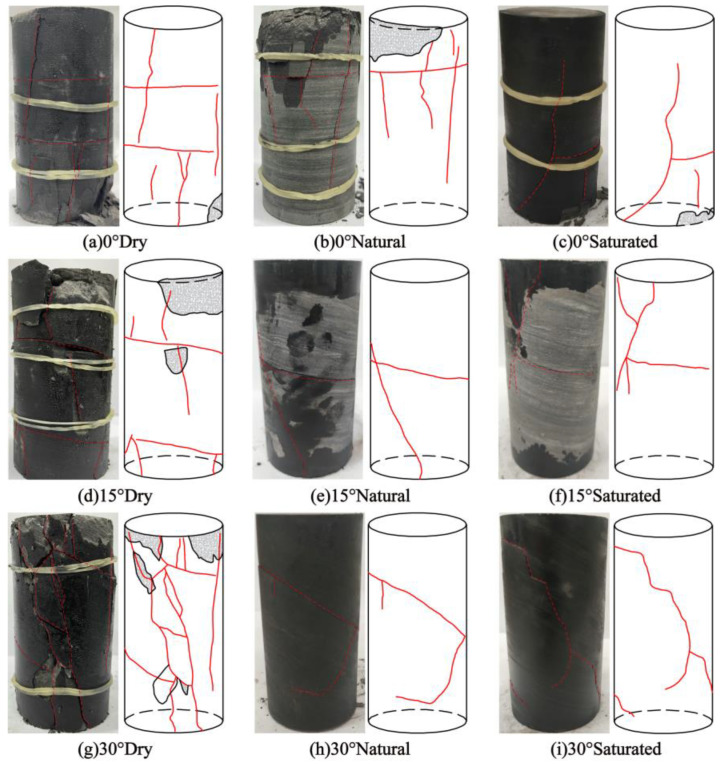
Crack propagation patterns of specimens with different water content and structural plane angles.

The macroscopic crack propagation morphology of layered shale samples, which are slightly inclined and have different structural plane angles and water content, under uniaxial compression is shown in Figure 4 (the left figure shows the final state of the specimen and the right figure shows the crack schematic diagram). By comparing the failure characteristics of the layered shale samples, it can be found that there is a substantial link between the crack propagation morphology of the samples under conditions of uniaxial compression and the incline angle and the water content of the structural plane. When the structural plane intersects with the original tilt layer at an angle of 0° and the specimen is subjected to axial loading, a vertical crack emerges at the center of the specimen. With an increase in load, the specimen undergoes shear failure, resulting in the formation of numerous cracks along the plane of the tilt. Cracks intersect with vertical cracks, then propagate and intrude, eventually forming a tensile failure pattern that is roughly along the axial load direction. For structural plane angles of 15° and 30°, the sample shows varying degrees of obliquity penetrating cracks, showing typical fracture characteristics of tensile-shear composites. This is primarily due to the fact that there is a large angle between the loading direction and the structural plane, and there will be relative sliding of the structural plane inside the specimen under vertical loading.

In addition, by comparing samples with the same structural plane angle, as can be seen, the moisture content of the sample has a significant impact on the crack count. As the moisture content increases, the number of accompanying cracks on the sample surface increases progressively, and cracks that lie roughly along the loading direction continue to grow and connect to the circumferential cracks. In some samples, surface peeling and severe damage are observed, with the 30° saturated sample being the most prominent.

**Figure 5:**
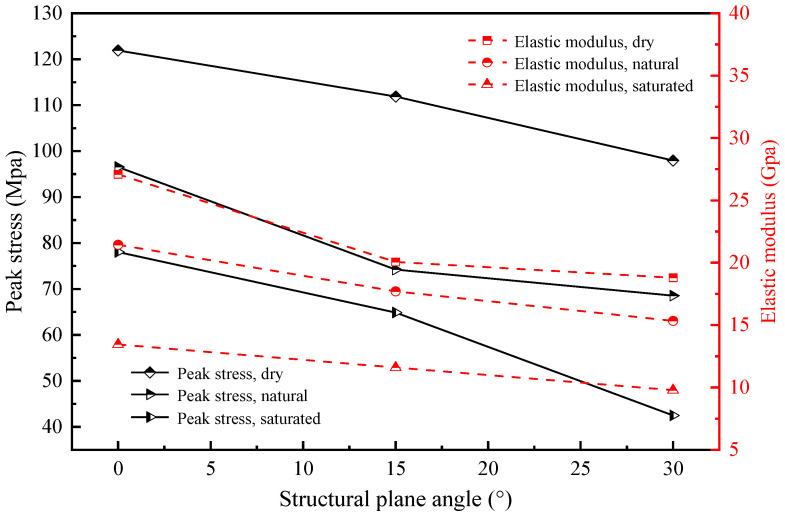
Variation curves of peak stress and elastic modulus with structural plane angle for specimens with different water content.

Figure 5 illustrates the variations in the peak uniaxial compressive strength and elastic modulus of distinct specimens with respect to the structural plane angle and moisture content. The outcomes reveal that the dry sample and 0° structural plane angle exhibit the highest uniaxial compressive strength (121.90 MPa) and elastic modulus (27.09 GPa). In contrast, when the structural plane angle and moisture content increase, the uniaxial compressive strength (42.46 MPa) and elastic modulus (9.78 GPa) of the saturated specimen at the 30° structural plane angle reaches the lowest value compared to the other samples.

Alternatively, it can be inferred that a linear decrease in both the uniaxial compressive strength and the elastic modulus occurs as the angle of the structural plane increases, provided that the moisture content of the sample remains constant. For instance, in the case of the natural water content sample, a rise in the angle of the structural plane from 0° to 30° resulted in a decrease in the uniaxial compressive strength from 96.53 MPa to 68.54 MPa, and a decrease in the elastic modulus from 21.44 GPa to 15.34 GPa. Assuming a constant angle of the structural plane, the uniaxial compressive strength and elastic modulus of the sample exhibit a decreasing trend with an increase in water content. For instance, in the case of a sample with a structural plane angle of 15°, the compressive strength and elastic modulus decreased by 42.0% and 42.2%, respectively, when the moisture content increased from dry to saturated. Analysis of the peak stress and elastic modulus variation characteristics indicates that the strength of the layered shale exhibits a negative correlation with the angle of the structural planes and the water content.

### 4.2. Analysis of Acoustic Emission Characteristic Parameters

#### 4.2.1. AE Ringing Counts

**Figure 6:**
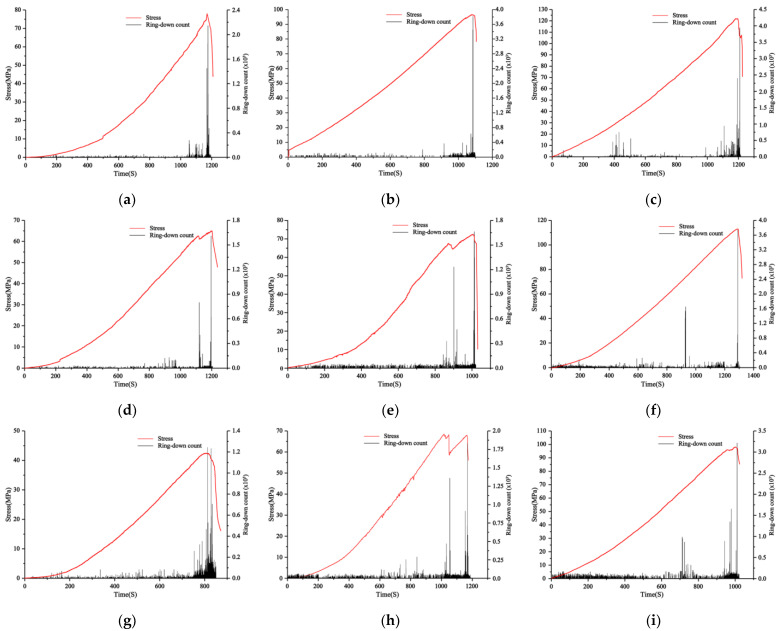
Acoustic emission ringing count and stress over time curve of shale sample: (**a**) 0° Saturated; (**b**) 0° natural; (**c**) 0° dry; (**d**) 15° saturated; (**e**) 15° natural; (**f**) 15° dry; (**g**) 30° saturated; (**h**) 30° natural; (**i**) 30° dry.

The AE counts refer to the frequency of AE signal excursions exceeding the AE threshold. This metric serves as an indicator of the extent of AE activity within the tested material or structure. Figure 6 shows the acoustic emission ringing number and stress versus time plots of shale samples with different water content and different tilt angles of the structural planes. From Figure 6, it can be observed that the generation of shale’s acoustic emission ringing counts follows a similar pattern under various conditions. Most of the time, the ringing counts are relatively small. Nonetheless, at the threshold of the rock’s ultimate stress capacity, both the frequency and intensity of the acoustic vibrations attain their highest amplitudes. Additionally, during stress discontinuities, there is an instantaneous increase in the frequency and magnitude of the ringing counts.

Based on the morphology of crack propagation and the values of the acoustic emission feature during specimen loading, the damage evolution process of gently inclined layered shale under uniaxial compression can be roughly divided into four stages:

(1) Initial compaction stage: During the initial phase of external loading on a layered shale specimen, the specimen undergoes an initial state of compaction. At this stage, the AE events primarily result from the internal compaction of the pores or microcracks.
(2) Micro crack compression stage: As the external load is further increased, the micro pores or cracks are compacted, and the number of AE events rises sharply. While the number of AE events gradually coalesces close to the plane of the structure, the structural plane gradually becomes the main area of crack and damage distribution, at which time the cracks appear as small cracks.
(3) Crack propagation stage: As loading is continued to be applied, the cracks gradually recur and propagate and the degree of damage accumulation in the attachment of the structural plane increases further.
(4) Crack penetration stage: When the load reaches 100%, the crack continues to grow and penetrates the entire sample of rock. The acoustic emission event reaches its peak value throughout the process, and the sample undergoes failure along the structure plane.

Upon observation of the loading process, a discernible trend can be observed whereby the total number of AE events increases gradually as the external load is progressively augmented. The occurrence of AE events represents the occurrence of damage at that location. As the external loads persist, internal damage and microcracks within the rock sample continue to accumulate and expand, eventually culminating in the deformation and ultimate failure of the layered shale sample.

A comparison of samples with different moisture contents reveals that when the structural plane tilt angle is the same, as the moisture content of the sample increases, the rate of acoustic emission events gradually decreases, regardless of the entire loading step or the instantaneous failure of the sample. This is because water can cause damage to the sample, leading to less energy being accumulated, dissipated, and released throughout the loading process.

A comparison of samples with different tilt angles in the structural plane indicates that when the moisture content is the same, the rate of AE events gradually increases as the tilt angle of the sample increases. This is attributed to the decrease in the overall strength of the rock resulting from the increased tilt angle of the shale, as well as the reduction in the compaction stage of the rock. Consequently, as the specimen is subjected to loading, the occurrence of internal crack damage intensifies, causing an increase in the rate of AE events. Meanwhile, it can be observed that the peak strength of the rock decreases as the tilt angle increases. This is because the maximum ringing count represents the rock’s peak strength. The increase in tilt angle results in a decrease in the peak strength of the rock, consequently leading to a reduction in the maximum ringing count.

#### 4.2.2. AE Energy Characteristics

**Figure 7:**
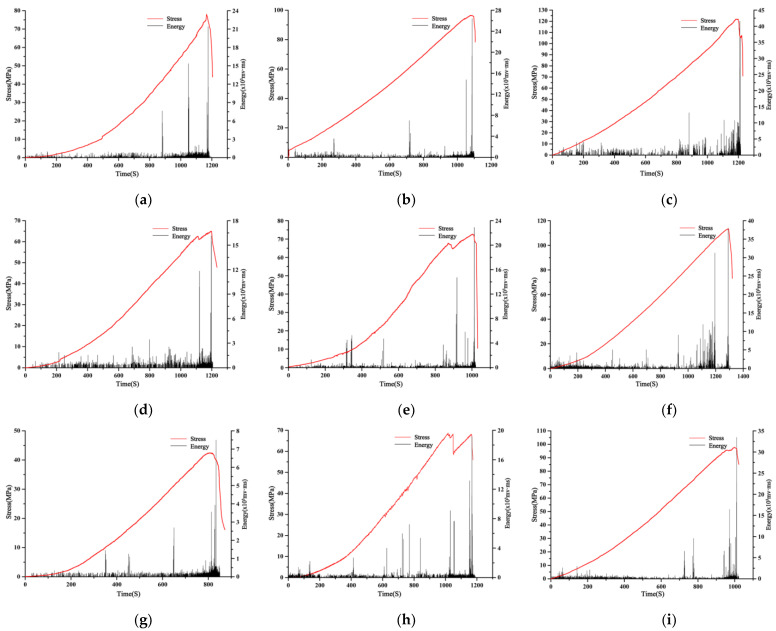
Curve of acoustic emission energy and stress of shale samples over time: (**a**) 0° Saturated; (**b**) 0° natural; (**c**) 0° dry; (**d**) 15° saturated; (**e**) 15° natural; (**f**) 15° dry; (**g**) 30° saturated; (**h**) 30° natural; (**i**) 30° dry.

We can think of the rock fracture process as a problem of non-equilibrium phase transition from a stationary to an unstable state, and the point of transition between them is called the critical point [42]. Acoustic emission information not only reflects the mechanical properties of the rocks but also contains information about the progress of internal damage in the rocks. The time instant of the abrupt change in AE events caused by crack propagation and penetration can be considered the critical point of instability of the specimen during the loading process [42,43,44]. Among these, the energy of acoustic emission can reflect the energy accumulation and release laws of the sample. Figure 7 displays the variations in AE energy and stress in shale samples under different loading conditions, comprising distinct water content and tilt angles.

From Figure 7, it can be observed that the energy variation pattern of shale is generally consistent across different conditions. Most of the time, the energy release is relatively small. However, near the maximum stress value of the rock, the energy release reaches its maximum value. Additionally, during stress discontinuities, there is an instantaneous increase in the energy release. An increase in the water content of the sample as well as in the angle of the structural plane will result in a corresponding decrease in rock strength and a lower amount of energy released. During the sample loading process, there will be more than one absolute energy mutation zone, which is consistent with the initiation, evolution, and temporal distribution of the main fracture surface. In general, the higher the relative displacement rate of the main fracture surface, the greater the release of energy. As the water content increases, the relative rate of displacement of the main rupture surface gradually decreases throughout the entire rupture, indicating that water reduces the energy storage capacity of the sample thereby reducing the severity of deformation and failure of the sample.

It is essential to note that the difference in the value and the degree of the energy released from the sample can be closely related to the sample failure mode under load. During the initial loading step, internal pores or microcracks within the sample are compacted, and the energy liberated accompanied by acoustic emission during that phase is relatively uniform; The continued increase in external load leads to further compaction of pores or microcracks, cracks and damage start to appear near the structural surface, and the release of acoustic emission energy begins to increase instantaneously; As the load is progressively augmented, the propagation of cracks is sustained, resulting in shear failure along the structure’s plane or splitting failure, which is accompanied by crack penetration, leading to a significant surge in the rock’s acoustic emission energy. Hence, while scrutinizing the engineering properties of tunnel envelopment in gently inclined bedded shale formations, it is crucial to account for the shale’s structural characteristics and the impact of moisture content.

### 4.3. Crack Classification Analysis

#### 4.3.1. Classification Theory

The primary process of rock failure is the initiation, spreading, and penetration of internal microcracks. The main types of rock failure include tensile and shear failures, and the proportion of them varies continuously with the progressive loading. Therefore, it is of great importance to explore the changes between the two during each loading step to understand the failure mechanism of gently tilt layered shale and to guide engineering practice.

**Figure 8:**
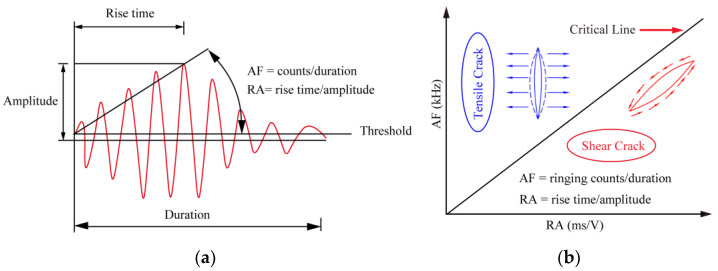
Acoustic emission crack classification theory: (**a**) Typical AE waveform; (**b**) micro cracks classification.

Based on the parameter analysis method described in the JCMS-IIIB5706 standard [45], tensile and shear cracks can be differentiated by utilizing key parameters such as RA and AF. The concept of “amplitude”, as defined by Grosse and Ohtsu [46], refers to the maximum amplitude present in a waveform, while “rise time” pertains to the duration between the initial signal and the peak amplitude. The “duration” is identified as the time range from the first point to the last point at which the shock wave crosses the threshold, and “count” refers to the number of times the signal amplitude crosses the threshold, as illustrated in Figure 8a. RA is the ratio of the rise time of acoustic emission to the amplitude of acoustic emission, in ms·v^−1^. Average Frequency (AF) refers to the ratio of the number of rings to the duration, measured in kHz. The relative relationship between the RA and AF values may therefore reflect the type of internal failure of the crack in the material. The slope of the divider line, denoted as ‘k’, is also defined. A signal is considered a shear failure signal when it has an AF/RA ratio less than k, according to Figure 8b.

#### 4.3.2. Crack Classification Based on AE Parameters

**Figure 9:**
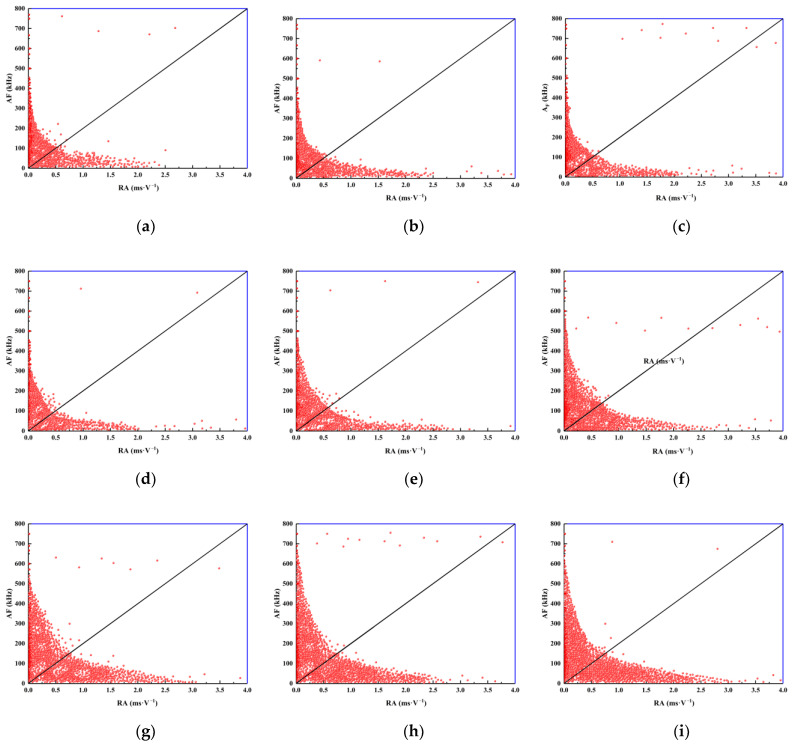
Scatter distribution of RA-AF in shale with different water content and tilt angles: (**a**) 0° saturated; (**b**) 0° natural; (**c**) 0° dry; (**d**) 15° saturated; (**e**) 15° natural; (**f**) 15° dry; (**g**) 30° saturated; (**h**) 30° natural; (**i**) 30° dry.

The parameters of the acoustic emission time series, comprising rise time, maximum amplitude, ringing count, and duration, for various shale samples are subjected to statistical analysis during the loading process. The objective of this analysis is to determine the values of RA and AF. The value of k is determined from this via the inversion of the moment tensor, and two regions of tensile and shear failure are correspondingly partitioned. Scatter plots of RA and AF data from different samples are shown in Figure 9. The global features of the rock samples’ RA-AF scatter plots under various working conditions demonstrate that high RA values correspond to low AF values, while low RA values correspond to high AF values, as shown in Figure 9. The distinction between tensile and shear crack fracture mechanisms is an important acoustic characterization, indicating that tensile as well as shear failures occur in the process of compressive uniaxial failure of samples with different tilt angles and water contents. Taking the rock samples with a structural plane inclination angle of 0° as an example, most of the RA-AF data points are distributed close to the AF axis, mostly concentrated in the tensile crack zone, and a small number of data points fall close to the axis of RA, which corresponds to the zone of shear cracks. This indicates that the fracture of rocks with three water contents at a tilt angle of 0° results in tensile and shear cracks, with tensile cracks being the main type of fracture.

The present study employs the crack classification method proposed by JCMS-IIIB5706 to classify cracks observed in shale uniaxial compression tests conducted with varying tilt angles and water content. Table 1 provides statistical information pertaining to the proportion of tensile and shear failure of shales with different water contents and tilt angles. The data presented in Table 1 indicate a strong correlation between the failure mode of the sample and the tilt angle and moisture content of the structure plane.

When the tilt angle is set to 0°, the majority of samples experience tensile failure, with a proportion exceeding 60%. This phenomenon is attributed to the axial loading direction of the sample being perpendicular to the tilt direction, causing compressive and expansive forces to act on each tilt. As a result, vertical cracks form at the center of the sample and propagate outward due to the tensile failure. Due to the rapid propagation of cracks in the center of the sample and the slow propagation of cracks at each end, shear dislocations form between layers, and large numbers of cracks initiate in the cross-sectional direction of the sample. This part of the crack propagates and forms transverse shear-breaking cracks.

For 15°and 30° tilt angles, some specimens have a slightly greater degree of shear than tensile failure. The proportion of shear and tensile failure in specimens is generally equivalent, manifesting itself in the form of composite tensile-shear failure. The main reason for this is the axial loading leading to the initiation of a large number of cracks on the tilt plane of the specimen. This portion of the cracks continuously initiates and dilates as the load is increased, causing shearing of the sample and movement along the tilt plane. Finally, at the two ends of the sample, cracks along the tilt plane intersect with the vertical cracks, forming the final composite tensile-failure form of the specimen. In addition, by comparing specimens with the same angle of tilting of the structural plane, they found that as the water content increases, the proportion of data points showing shear failure in rock samples gradually decreases. These results indicate that under uniaxial loading conditions, the higher the water content, the greater the tensile failure that occurs in the rock samples.

From the experimental observations, we can conclude that the failure characteristics reflected by the distribution of the RA-AF acoustic emission values are highly consistent with the crack propagation morphology of the sample, indicating that the acoustic emission values from RA-AF can effectively characterize the crack propagation mode of the specimen. From the RA-AF value of the acoustic emission, it is possible to accurately determine the relationship between the tilt angle, water content, and crack propagation mode of the layered shale, which is of great importance for revealing the crack propagation and failure mode of layered shales with different tilt angles and water content.

Based on the results of the acoustic emission (AE) tests, it can be concluded that in the Chaoyang Tunnel, the rock mass with higher water content and larger tilt angles (0°~30°) in the gently inclined layered shale section exhibits poorer load-bearing capacity. Consequently, the construction risk of the tunnel increases. Therefore, in the design and construction process, more secure structural and construction methods have been adopted to effectively control the deformation of the tunnel structure.

## 5. Conclusions

This study conducted uniaxial compression tests on prefabricated gently sloping layered shale specimens with varying structural plane angles and water content. An AE monitoring system was employed to analyze the damage evolution process of the specimens, investigate the crack extension mechanism, and examine the time-series variation characteristics of acoustic emission parameters during rock damage. The study yielded the following main conclusions:

1. The study of the damage evolution process of rock samples under uniaxial compression conditions can be effectively conducted through the implementation of acoustic emission counting and energy analysis techniques. As the load increases, the number of acoustic emissions and the energy value continue to increase, representing the propagation of cracks from the rock specimen. The detection of peak acoustic emission counts and peak energy values in close proximity to peak stress levels can serve as precursors to the failure of the rock sample.
2. The study of the damage evolution process of rock samples under uniaxial compression conditions can be effectively conducted through the implementation of acoustic emission counting and energy analysis techniques. As the load increases, the number of acoustic emissions and the energy value continue to increase, representing the propagation of cracks from the rock specimen. The detection of peak acoustic emission counts and peak energy values in close proximity to peak stress levels can serve as precursors to the failure of the rock sample.
3. The study of the damage evolution process of rock samples under uniaxial compression conditions can be effectively conducted through the implementation of acoustic emission counting and energy analysis techniques. As the load increases, the number of acoustic emissions and the energy value continue to increase, representing the propagation of cracks from the rock specimen. The detection of peak acoustic emission counts and peak energy values in close proximity to peak stress levels can serve as precursors to the failure of the rock sample.

The study of the damage evolution process of rock samples under uniaxial compression conditions can be effectively conducted through the implementation of acoustic emission counting and energy analysis techniques. As the load increases, the number of acoustic emissions and the energy value continue to increase, representing the propagation of cracks from the rock specimen. The detection of peak acoustic emission counts and peak energy values in close proximity to peak stress levels can serve as precursors to the failure of the rock sample.

A noteworthy relationship has been observed between the failure morphology of gently tilted layered shale and the angle and water content of the structural planes of the rock mass. When the angle of the structural plane of the rock sample is small (0°), tensile failure primarily takes place. The increase in the structural plane angle (15°, 30°) results in a gradual transition from failure mode to composite tensile-shear failure. As the water content increases, the degree of damage to the rock sample increases.

From the RA-AF values of acoustic emission, it is possible to accurately determine the relationship between the tilt angle, water content, crack propagation mode, and failure morphology of gently tilt-layered structural planes of shale. The tilt angle of the structural plane is the primary factor influencing the shape of the failure for rock samples. In addition, changes in water content can also affect the failure characteristics of rock samples to some extent.

## Figures and Tables

**Table 1:** Proportion of tensile failure and shear failure of rock samples under uniaxial compression conditions.

Working Condition	Compression Strength (MPa)	Proportion of Tension Failure (%)	Proportion of Shear Failure (%)	Main Failure Mode
0° Saturated	78.0	68.2	31.8	Tensile failure
0° Natural	96.5	65.8	34.2	Tensile failure
0° Dry	121.9	60.2	39.8	Tensile failure
15° Saturated	64.9	58.7	41.3	Tensile failure
15° Natural	74.2	53.2	46.8	Composite tensile-shear failure
15° Dry	111.9	51.4	48.6	Composite tensile-shear failure
30° Saturated	42.5	50.5	49.5	Composite tensile-shear failure
30° Natural	68.5	47.8	52.2	Composite tensile-shear failure
30° Dry	98.0	44.7	55.3	Composite tensile-shear failure

## Data Availability

Not applicable.
